# Population-Scale Plasma Proteomic Profiles Associated with Chronic Periodontitis in the UK Biobank

**DOI:** 10.3390/ijms27052514

**Published:** 2026-03-09

**Authors:** Su Kang Kim, Min Kyoung Kim, Sang Wook Kang, Ju Yeon Ban

**Affiliations:** 1 Department of Biomedical Laboratory Science, Catholic Kwandong University, Gangneung 25601, Republic of Korea; skkim7@cku.ac.kr; 2 Department of Dental Pharmacology, College of Dentistry, Dankook University, Cheonan 31116, Republic of Korea; rlaalsrud010330@naver.com; 3 Department of Oral and Maxillofacial Pathology, College of Dentistry, Kyung Hee University, Seoul 02447, Republic of Korea

**Keywords:** chronic periodontitis, plasma proteomics, Olink Explore, systemic inflammation, GDF15, IL-6, TNF receptor superfamily, biomarkers, UK Biobank

## Abstract

Periodontitis is a chronic infectious disease characterized by the destruction of the tooth-supporting tissues, including the gingiva, periodontal ligament, and alveolar bone, which may ultimately lead to tooth loss. However, blood-based biomarkers reflecting systemic inflammation in periodontitis remain poorly defined. We analyzed plasma proteomic data from the UK Biobank using Olink Explore proteomics to identify systemic protein signatures distinguishing chronic periodontitis patients (*n* = 90) from healthy controls (*n* = 2234). Among 2151 proteins passing quality control, 29 proteins showed significant differential expression (FDR < 1.0 × 10^−5^). Growth differentiation factor 15 (GDF15) exhibited the strongest upregulation (mean NPX: −0.183 to 0.157, effect size = 0.337, FDR = 2.82 × 10^−12^), followed by N-terminal pro-B-type natriuretic peptide (NT-proBNP) (effect size = 0.594), Interleukin-6 (IL-6) (effect size = 0.450), and Insulin-like growth factor binding protein-(4IGFBP4) (effect size = 0.269). Multiple TNF receptor superfamily members (TNFRSF1A/1B, TNFRSF10A/10B) and proteins involved in extracellular matrix remodeling (COL6A3, ADAM12) and vascular stress (ADM) were significantly elevated. In contrast, EGFR and DNER showed decreased expression. Protein–protein interaction network analysis revealed IL-6 as a central hub protein forming a tightly interconnected cluster with TNF receptor family members. These findings indicate systemic plasma protein profiles associated with chronic periodontitis within this population-based cohort. The identified proteins may provide a basis for future evaluation of blood-based biomarkers for chronic periodontitis, pending further validation.

## 1. Introduction

Chronic periodontitis is a common chronic infectious disease characterized by the progressive destruction of the tooth-supporting tissues, including the gingiva, periodontal ligament, cementum, and alveolar bone, which may ultimately result in tooth loss [1]. The loss of periodontal support and subsequent tooth loss substantially impair masticatory function, nutritional intake, social interaction, and overall quality of life [2].

The initiation and progression of chronic periodontitis are primarily driven by a dysbiotic subgingival biofilm and dysregulated host immune–inflammatory responses [3,4]. In addition, several major systemic conditions, including diabetes mellitus, cardiovascular disease, obesity, and chronic obstructive pulmonary disease, have been reported to be associated with the clinical course of periodontitis and represent important clinical background factors when considering the potential systemic relevance of periodontal disease [5].

Clinically, chronic periodontitis is diagnosed and its severity is assessed using established local parameters, including probing depth, clinical attachment loss, bleeding on probing, and radiographic evaluation of alveolar bone loss. According to contemporary classification systems, periodontitis is further staged and graded to reflect disease severity and risk of progression [6]. However, in large population-based cohorts derived from electronic health records, detailed clinical information required for staging and grading is often unavailable, which limits precise stratification of disease severity.

At the molecular and cellular levels, periodontal tissue destruction is mediated by multiple interacting pathogenic mechanisms, including Toll-like receptor signaling, dysregulated neutrophil responses, complement activation, extracellular matrix degradation, epithelial barrier dysfunction, and osteoclast-mediated bone resorption [7,8]. Although these mechanisms have been extensively investigated in experimental and clinical studies, how such diverse pathological pathways are integrated and reflected at the level of the systemic circulating proteome remains incompletely understood.

In routine clinical practice, periodontitis can be readily identified and monitored using well-established local clinical indices. Therefore, investigation of circulating protein alterations associated with periodontitis should be regarded as complementary to clinical diagnosis, providing additional biological insights into the systemic responses accompanying local periodontal inflammation rather than serving as a diagnostic substitute [9]. Recent large-scale plasma proteomic studies have similarly highlighted the value of multiplex circulating protein signatures for disease risk prediction in population-based cohorts [10].

Therefore, in this study, we performed a population-based comparative analysis of plasma protein profiles between individuals with chronic periodontitis and normal controls using large-scale cohort data. Our aim was to characterize systemic protein alterations associated with chronic periodontitis and to explore biological pathways potentially involved in the systemic manifestations of the disease.

## 2. Results

### 2.1. Overall Differences in Plasma Protein Expression Between the Normal and Chronic Periodontitis Groups

**Figure 1:**
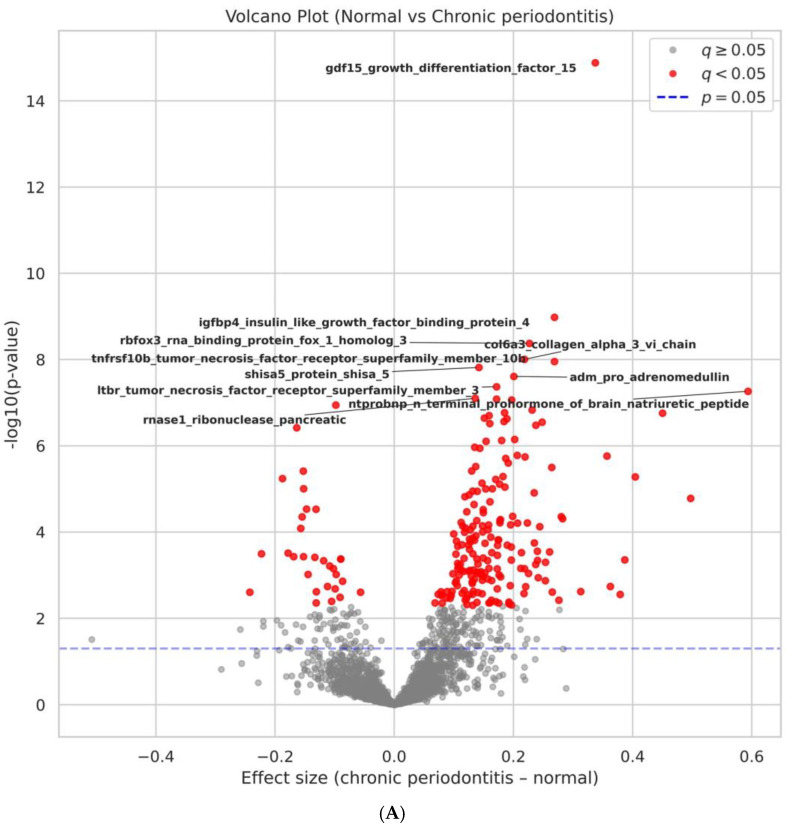

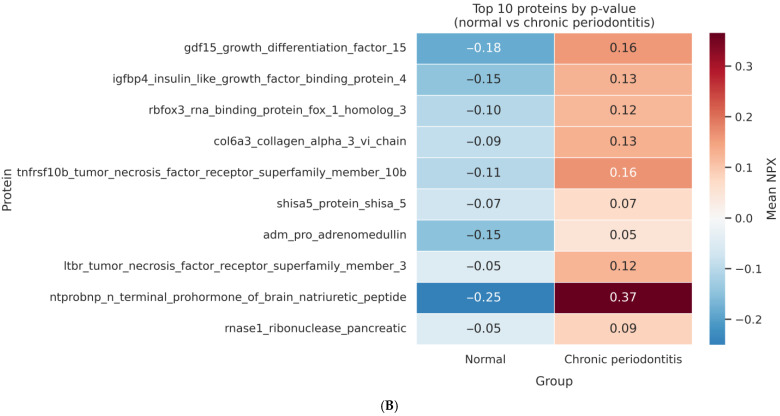
Volcano plot and heatmap of differentially expressed plasma proteins between the normal and chronic periodontitis groups. (**A**) Volcano plot showing effect size and statistical significance of plasma proteins. Red dots indicate proteins with FDR < 0.05. (**B**) Heatmap of the top 10 differentially expressed proteins based on mean normalized protein expression (NPX) values in each group. Red indicates higher NPX values, whereas blue indicates lower NPX values.

A total of 2151 proteins passed all quality control (QC) criteria. Comparison of plasma protein expression between the normal and chronic periodontitis groups identified statistically significant differential expression across multiple proteins (Figure 1). For each protein, the mean values in the normal and chronic periodontitis groups, effect size, *p*-value, sample size (*n*), and false discovery rate (FDR) were calculated. Statistical significance was defined using an FDR threshold of <0.05. Overall, the chronic periodontitis group exhibited higher expression levels for numerous proteins compared with the normal group, whereas a subset of proteins showed reduced expression.

Among the differentially expressed proteins, GDF15 exhibited the most significant differential expression. In addition, IGFBP4, RBFOX3, COL6A3, TNFRSF10B, SHISA5, ADM, LTBR, NT-proBNP, and RNASE1 were also elevated in the chronic periodontitis group. Volcano plot analysis (Figure 1A) demonstrated that proteins upregulated in the chronic periodontitis group displayed positive effect sizes with high statistical significance, whereas proteins with relatively higher expression in the normal group exhibited negative effect sizes. The normal and chronic periodontitis groups showed a clear separation in their overall protein expression profiles.

Heatmap analysis of the top 10 differentially expressed proteins (Figure 1B) further demonstrated distinct expression patterns between the two groups. Most proteins in the normal group exhibited relatively low expression levels, whereas many proteins showed consistently higher expression in the chronic periodontitis group. In contrast, EGFR displayed a decrease in expression in the chronic periodontitis group compared with the normal group.

### 2.2. Key Proteins Significantly Altered in the Chronic Periodontitis Group

Table 1 summarizes the results of the differential protein abundance analysis between healthy controls and patients with chronic periodontitis (FDR < 1.0 × 10^−5^). The most significantly increased protein in the chronic periodontitis group was GDF15, which increased from a mean value of −0.183 in the normal group to 0.157 in the chronic periodontitis group, corresponding to an effect size of 0.337 and an FDR of 2.82 × 10^−12^. IL-6 also showed a marked increase, from −0.128 in the normal group to 0.324 in the chronic periodontitis group (effect size = 0.450, FDR = 2.35 × 10^−5^). IGFBP4 increased from −0.146 to 0.127 (effect size = 0.269, FDR = 1.13 × 10^−6^), and NT-proBNP increased from −0.250 to 0.366 (effect size = 0.594, FDR = 1.30 × 10^−5^).

Significant increases were also observed in several members of the tumor necrosis factor (TNF) receptor family, including TNFRSF1A, TNFRSF1B, TNFRSF10A, and TNFRSF10B. In addition, multiple other proteins were significantly elevated in the chronic periodontitis group compared with the normal group, including COL6A3, ADM, LTBR, LGALS9, VSIG4, FSTL3, RNASE1, NPC2, COLEC12, ADAM12, LAIR1, CST3, PRAP1, SORCS2, SCARB2, PGF, and RNASE6.

In contrast, a small number of proteins exhibited decreased expression in the chronic periodontitis group. EGFR decreased from a mean value of 0.048 in the normal group to −0.050 in the chronic periodontitis group (effect size = −0.098, FDR = 1.88 × 10^−5^). Similarly, DNER decreased from 0.086 in the normal group to −0.080 in the chronic periodontitis group, with an effect size of −0.164 and an FDR of 3.43 × 10^−5^.

### 2.3. Distribution Analysis of the Top 10 Differentially Expressed Proteins

**Figure 2:**
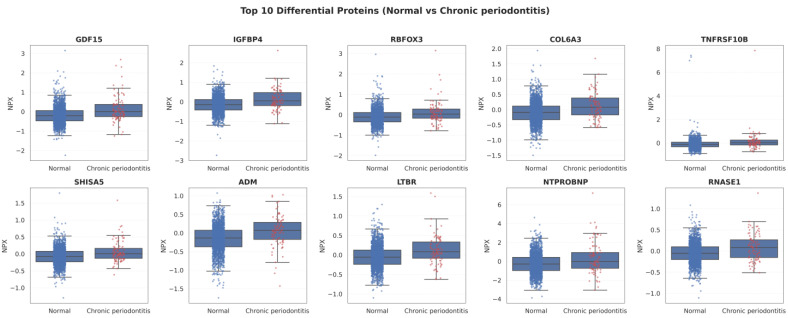
Boxplots of the top 10 differentially expressed plasma proteins between the normal and chronic periodontitis groups. Boxplots show the distribution of normalized protein expression (NPX) values for GDF15, IGFBP4, RBFOX3, COL6A3, TNFRSF10B, SHISA5, ADM, LTBR, NT-proBNP, and RNASE1.

Figure 2 illustrates the distribution of the top 10 differentially expressed proteins between the normal and chronic periodontitis groups using boxplot analysis. The analysis was performed for the following proteins: GDF15, IGFBP4, RBFOX3, COL6A3, TNFRSF10B, SHISA5, ADM, LTBR, NT-proBNP, and RNASE1. For all ten proteins, the median normalized protein expression (NPX) values were consistently higher in the chronic periodontitis group than in the normal group.

In addition, for several proteins, including GDF15, IGFBP4, RBFOX3, COL6A3, and NT-proBNP, the upper quartiles (Q3) and high value outliers were more frequently observed in the chronic periodontitis group. For some proteins, the overlap in distribution between the two groups was limited, and notably, GDF15, NT-proBNP, and TNFRSF10B exhibited clearly distinct distribution patterns between the normal and chronic periodontitis groups. Although extreme values were present in both groups for certain proteins, such as TNFRSF10B and NT-proBNP, the chronic periodontitis group demonstrated a wider range of high-value distributions.

Overall, while the distributions of the normal and chronic periodontitis groups were not completely separated, a consistent upward shift in NPX values was observed across all ten proteins in the chronic periodontitis group.

### 2.4. Protein Expression Changes by Olink Panel

**Figure 3:**
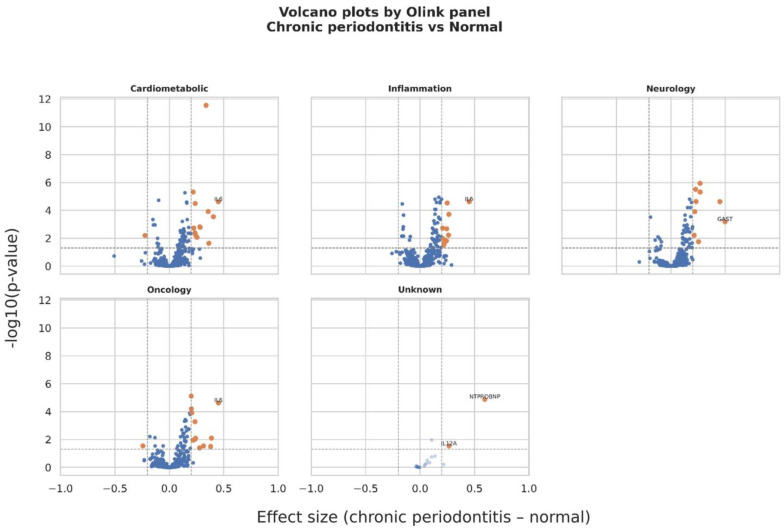
Panel-specific volcano plots of plasma protein expression differences between the normal and chronic periodontitis groups. Volcano plots are shown for the cardio-metabolic, inflammation, neurology, oncology, and unknown Olink panels. Orange dots indicate significantly differentially expressed proteins, whereas blue dots indicate non-significant proteins. Vertical dashed lines indicate the effect size threshold, and the horizontal dashed line indicates the statistical significance threshold.

Figure 3 presents the results of the volcano plot analysis stratified by Olink panel, including cardio-metabolic, inflammation, neurology, oncology, and unknown categories [11]. Across all panels, multiple proteins exhibited significantly positive effect sizes, indicating higher expression levels in the chronic periodontitis group compared with the normal group. IL-6 was consistently identified as a significantly increased protein across several panels. In the neurology panel, GAST displayed a significant positive effect size, while NT-proBNP showed the largest positive effect size with high statistical significance in the unknown panel. In contrast, only a limited number of proteins exhibited significantly negative effect sizes.

### 2.5. Protein–Protein Interaction (PPI) Network Analysis

**Figure 4:**
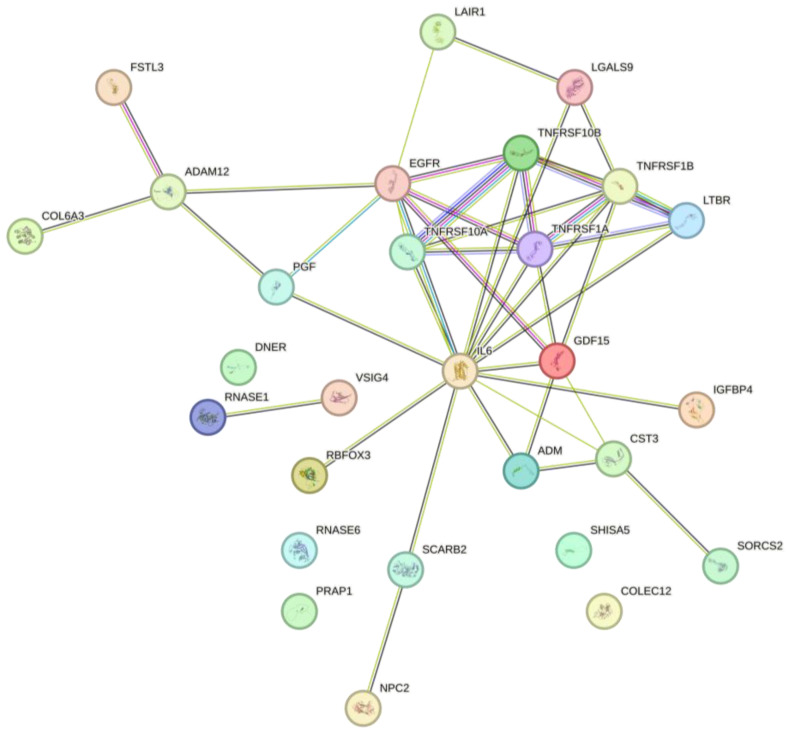
STRING-based protein–protein interaction (PPI) network of significantly altered proteins between the normal and chronic periodontitis groups. The network illustrates functional interactions among differentially expressed plasma proteins. The colored lines represent different types of evidence supporting the interaction: **green** indicates gene neighborhood, **red** indicates gene fusion, **blue** indicates gene co-occurrence, **purple** indicates experimentally determined interactions, **yellow** indicates text-mining evidence, **light blue** indicates database annotations, and **black** indicates co-expression. The thickness of the edges reflects the confidence score of the interaction.

Figure 4 illustrates the protein–protein interaction network of the differentially expressed proteins [12]. IL-6 was identified as the central hub protein with the highest number of interactions. Members of the TNF receptor superfamily (TNFRSF1A, TNFRSF10A, TNFRSF10B, and TNFRSF1B) formed a tightly interconnected core cluster together with LTBR, LGALS9, and LAIR1. GDF15 was located near the central hub and was directly connected to several core proteins. A distinct branch consisting of COL6A3, ADAM12, and FSTL3 was observed, representing an independent subnetwork. Additional peripheral nodes, including ADM, PGF, SCARB2, RNASE1, RBFOX3, and VSIG4, were connected to the central network with fewer interactions.

## 3. Discussion

The concept of chronic periodontitis, previously regarded as a localized infection driven by excessive plaque accumulation, has evolved toward an immune-mediated disease characterized by microbial dysbiosis and dysregulated host immune responses [3]. Under this framework, local periodontal inflammation may be accompanied by broader systemic immune and metabolic alterations. However, how such local pathological processes are reflected in circulating plasma protein profiles at the population level remains incompletely understood. Recent molecular network-based analyses further support the systemic integration of periodontal inflammation with broader disease pathways [13].

Using population-scale plasma proteomic data, the present study identified proteins and protein networks associated with chronic periodontitis compared with the normal group. Consistent with previous clinical study reporting elevated circulating inflammatory markers in periodontitis, including IL-6 and TNF-related molecules, we observed significant increases in IL-6 and several TNF receptor family members in plasma [14]. Our findings extend these earlier observations by demonstrating, at the proteome-wide level, coordinated changes in multiple inflammation-related proteins within a large population-based cohort.

Among the altered proteins, GDF15 showed the largest effect size and the strongest statistical significance. Previous studies have reported that circulating GDF15 is associated with cellular stress, mitochondrial dysfunction, ageing, and chronic inflammatory condition [15,16,17]. To date, however, evidence regarding its role in periodontal disease has been limited. The present population-scale analysis suggests that GDF15 is associated with systemic inflammatory and stress-related responses in individuals with chronic periodontitis. Furthermore, the network-based analysis indicated that GDF15 was connected to IL-6 and members of the TNF receptor family, supporting a potential involvement of this protein in broader inflammatory response pathways. These network-level observations should be interpreted as hypothesis-generating and do not imply causal regulatory relationships.

In addition to inflammatory proteins, IGFBP4 was significantly increased in the chronic periodontitis group. IGFBP4 has been implicated in growth factor signaling and regulation of tissue microenvironment remodeling [18]. Although its involvement in periodontal inflammation has not been extensively investigated, our findings suggest that systemic alterations in growth factor-related pathways may accompany chronic periodontal inflammation.

Several extracellular matrix-related proteins, including COL6A3, ADAM12, and FSTL3, were also elevated. Previous experimental and clinical studies have demonstrated that extracellular matrix remodeling and metalloproteinase activity are key components of periodontal tissue destruction [19,20,21]. The concurrent elevation of these proteins in plasma may reflect systemic manifestations of tissue remodeling processes associated with periodontal inflammation, rather than being confined to local periodontal lesions.

We further observed elevated levels of NT-proBNP and ADM, which are well-established markers of cardiovascular and vascular stress responses [22,23]. Previous epidemiological studies have reported associations between periodontitis and cardiovascular diseases [24]. In this context, the present findings provide proteomic-level evidence supporting a molecular link between chronic periodontitis and systemic vascular or metabolic stress pathways. However, these associations should be interpreted cautiously, as comorbid cardiovascular conditions and medication use may also influence circulating levels of these proteins.

The network analysis further demonstrated that the identified proteins formed interconnected subnetworks related to inflammatory signaling, extracellular matrix remodeling, and vascular or stress responses. These network structures provide a systems-level overview of how multiple biological processes may be concurrently associated with chronic periodontitis. Nevertheless, these results are based on bioinformatics inference and should be considered exploratory.

The major strengths of this study include the large-scale population-based plasma proteomic analysis of chronic periodontitis using the UK Biobank, the use of Olink^®^ Explore NPX data accessed through the COMPASS™ platform, a rigorous definition of the normal group, and the application of a pathway-level, network-centric analytical approach. Together, these features enabled a robust characterization of systemic protein signatures associated with chronic periodontitis.

Several limitations should be acknowledged. Despite explicit adjustment for age and sex in all statistical models, the substantial imbalance in sample size between the chronic periodontitis group and the normal group, together with the relatively limited number of chronic periodontitis cases, may affect the stability of effect size estimates and increase sensitivity to residual confounding. In addition, periodontal phenotypes were defined based on diagnostic records and self-reported information, and detailed clinical periodontal parameters required for disease staging and grading were not available in the UK Biobank resource, precluding severity-based subgroup analyses. And the study population was restricted to individuals aged 40–69 years, and therefore the generalizability of the findings to younger or older populations should be interpreted with caution. This should be considered when interpreting the observed systemic protein alterations. Furthermore, this study was restricted to plasma proteomics without validation in saliva or gingival crevicular fluid, and the cross-sectional design limits inference regarding temporal relationships between periodontal status and systemic protein changes.

Future studies should aim to validate the identified protein signatures in non-invasive biological samples, including saliva and gingival crevicular fluid, and to evaluate their robustness in larger and more balanced study populations. In addition, targeted functional studies using cellular and animal models will be required to clarify the molecular regulatory pathways and causal roles of the core proteins identified in this study. Longitudinal investigations will also be needed to better characterize changes in systemic protein profiles in relation to periodontal treatment and disease progression.

## 4. Materials and Methods

### 4.1. Cohort Definition and Covariates

This study utilized plasma proteomic and clinical data from the UK Biobank, a large-scale prospective cohort comprising approximately 500,000 adults aged 40–69 years at recruitment. Analyses were restricted to participants with available plasma proteomic measurements generated using the Olink^®^ Explore platform. Phenotype assignment for chronic periodontitis was performed using the the web-based COMPASS™ platform (Cipherome Inc., San Jose, CA, USA) to identify individuals with a clinically recorded diagnosis of chronic periodontitis based on the ICD-10 code K05.3. Individuals classified as chronic periodontitis cases had a documented hospital diagnosis corresponding to ICD-10 code K05.3. Self-reported periodontal or gum disease fields, including UK Biobank field 131,562, were not used for case definition in this study. Normal controls were defined as individuals with no evidence of periodontal disease according to the same COMPASS-derived phenotype definitions. In addition, individuals with documented diabetes, hypertension, or dyslipidemia were excluded from the normal control group to minimize potential confounding effects of systemic comorbidities on plasma protein levels.

At the initial stage, a total of 2781 individuals were included, comprising 2676 normal controls and 105 individuals with chronic periodontitis. Proteomic measurements, sampling dates, and participant-level metadata were integrated using unique sample and participant identifiers to construct the analytical dataset. Age, sex, and the proteomic measurement date were included as covariates in all downstream regression analyses.

After quality control and exclusion of date-related outliers, a total of 2341 participants were included in the final analytical cohort, consisting of 2251 normal controls and 90 individuals with chronic periodontitis. The mean age was 69.0 ± 7.8 years in the normal control group and 69.1 ± 8.3 years in the chronic periodontitis group. In the chronic periodontitis group, 49 participants (54.4%) were female and 41 (45.6%) were male, whereas in the normal control group, 1163 participants (51.7%) were female and 1088 (48.3%) were male. The UK Biobank study was approved by the North West Multi-centre Research Ethics Committee, and this study was conducted under UK Biobank application number 52031.

### 4.2. UK Biobank Cohort and COMPASS™ Platform-Based Data Extraction

Plasma proteomic and clinical data were obtained from the UK Biobank under approved access. Participants with available Olink^®^ Explore plasma proteomic measurements were identified through the COMPASS™ platform, which was used as a data access and management interface to retrieve phenotype labels and participant-level metadata derived from multiple UK Biobank data sources. Proteomic measurements, periodontal phenotype labels, sampling dates, and proteomics-related technical metadata, including batch and plate information, were merged using unique participant and sample identifiers to construct the integrated analytical dataset.

### 4.3. Quality Control of Olink Proteomics and Metadata

Plasma protein abundance was measured using the Olink^®^ Explore platform and quantified as NPX values. Assay-specific limits of detection (LOD) provided by the platform were used for quality control. Each protein was matched to its corresponding assay-level LOD, and values below the LOD were flagged. Proteins for which more than 25% of samples had measurements below the LOD were excluded, resulting in 2174 proteins retained after protein-level LOD filtering. At the sample level, the proportion of measurements below the LOD was calculated across the retained proteins, and samples with more than 10% of values below the LOD were excluded; however, all samples satisfied this criterion and were retained at this step. Residual missingness was subsequently assessed. Proteins with more than 20% missing values were excluded, yielding a final set of 2151 proteins. Samples with more than 20% missing values across the retained proteins were also excluded, resulting in 2342 samples remaining after missingness-based filtering. This threshold was selected in accordance with commonly used quality-control practices in large-scale plasma proteomics studies to balance data completeness and protein coverage.

To identify potential technical outliers related to measurement dates, daily mean NPX values were calculated across all retained proteins, and Z-scores were computed for these daily averages. Dates with absolute Z-scores greater than 3 were defined as outliers, and one measurement date was removed. After exclusion of this outlier date, the final analytical dataset consisted of 2341 participants and 2151 proteins. To account for potential temporal effects in proteomic measurements, sample collection dates were converted into a continuous numeric variable representing the number of days since the earliest measurement date in the filtered dataset. This variable was included as a covariate in all downstream regression analyses.

### 4.4. Statistical Modeling and Differential Protein Analysis

To adjust for demographic and technical confounders, NPX values for each protein were regressed on age, sex, and numeric sample collection date using ordinary least-squares regression. The resulting residuals were interpreted as covariate-adjusted protein abundance values and were used for group-level comparisons. Differential protein abundance between individuals with chronic periodontitis and the normal group was evaluated using a linear modeling framework conceptually analogous to limma. For each protein, effect sizes defined as the mean difference in covariate-adjusted NPX values between groups were calculated, along with corresponding *p*-values and Benjamini–Hochberg FDR–adjusted q-values [25,26]. Proteins with FDR-adjusted q-values < 0.05 were considered statistically significant.

To explore proteomic patterns across biological domains, differential analyses were additionally performed within each Olink^®^ Explore panel category, including Inflammation, Cardiometabolic, Neurology, and Oncology. Results were visualized using volcano plots, heatmaps, and boxplots of top differentially abundant proteins. Volcano plots displayed effect sizes against −log_10_-transformed *p*-values, and heatmaps of covariate-adjusted NPX residuals were used to assess clustering patterns across participants. All analyses were conducted in Python (version 3.12, Python Software Foundation, Wilmington, DE, USA) using pandas, numpy, statsmodels, matplotlib, and seaborn within a fully reproducible computational workflow.

## 5. Conclusions

In summary, this population-based plasma proteomic analysis identified systemic protein alterations associated with chronic periodontitis, involving pathways related to inflammation, cellular stress, and tissue remodeling. These findings suggest that local periodontal inflammation is accompanied by measurable changes in circulating protein profiles and provide biological insights into potential links between periodontal disease and broader systemic responses. Further longitudinal and mechanistic studies will be required to clarify the clinical relevance and causal relationships of these systemic proteomic changes.

## Figures and Tables

**Table 1:** Differentially expressed plasma proteins between healthy controls and patients with chronic periodontitis in the UK Biobank Olink proteomics dataset.

Protein (Symbol_Full Name)	Mean NOR	Mean CP	Effect	*p* Value	*n* NOR	*n* CP	FDR
GDF15 (Growth differentiation factor 15)	−0.183	0.157	0.337	1.31 × 10^−15^	2223	89	2.82 × 10^−12^
IGFBP4 (Insulin-like growth factor-binding protein 4)	−0.146	0.127	0.269	1.05 × 10^−9^	2177	87	1.13 × 10^−6^
RBFOX3 (RNA binding fox-1 homolog 3)	−0.102	0.121	0.227	4.23 × 10^−9^	2225	90	3.03 × 10^−6^
COL6A3 (Collagen type VI alpha 3 chain)	−0.091	0.128	0.218	9.90 × 10^−9^	2223	89	4.76 × 10^−6^
TNFRSF10B (Tumor necrosis factor receptor superfamily member 10B)	−0.105	0.164	0.269	1.11 × 10^−8^	2222	87	4.76 × 10^−6^
SHISA5 (Shisa family member 5)	−0.073	0.069	0.142	1.52 × 10^−8^	2227	90	5.46 × 10^−6^
ADM (Adrenomedullin)	−0.152	0.050	0.200	2.46 × 10^−8^	2234	89	7.57 × 10^−6^
LTBR (Lymphotoxin beta receptor)	−0.048	0.123	0.172	4.27 × 10^−8^	2196	89	1.15 × 10^−5^
NT-proBNP (N-terminal pro-B-type natriuretic peptide)	−0.250	0.366	0.594	5.45 × 10^−8^	2212	86	1.30 × 10^−5^
RNASE1 (Ribonuclease A family member 1 (pancreatic ribonuclease))	−0.049	0.086	0.136	7.92 × 10^−8^	2226	89	1.55 × 10^−5^
TNFRSF1A (Tumor necrosis factor receptor superfamily member 1A)	−0.084	0.088	0.172	8.26 × 10^−8^	2208	88	1.55 × 10^−5^
LGALS9 (Galectin 9)	−0.116	0.084	0.197	8.67 × 10^−8^	2211	89	1.55 × 10^−5^
EGFR (Epidermal growth factor receptor)	0.048	−0.050	−0.098	1.14 × 10^−7^	2223	89	1.88 × 10^−5^
VSIG4 (V-set and immunoglobulin domain containing 4)	−0.127	0.106	0.231	1.48 × 10^−7^	2208	88	2.28 × 10^−5^
FSTL3 (Follistatin-like 3)	−0.104	0.083	0.185	1.70 × 10^−7^	2205	90	2.35 × 10^−5^
IL-6 (Interleukin 6)	−0.128	0.324	0.450	1.75 × 10^−7^	2212	89	2.35 × 10^−5^
NPC2 (NPC intracellular cholesterol transporter 2)	−0.081	0.080	0.159	1.99 × 10^−7^	2191	89	2.52 × 10^−5^
COLEC12 (Collectin subfamily member 12)	−0.090	0.064	0.151	2.28 × 10^−7^	2206	90	2.67 × 10^−5^
TNFRSF1B (Tumor necrosis factor receptor superfamily member 1B)	−0.084	0.106	0.189	2.36 × 10^−7^	2208	88	2.67 × 10^−5^
ADAM12 (ADAM metallopeptidase domain 12)	−0.048	0.140	0.184	2.73 × 10^−7^	2161	88	2.91 × 10^−5^
LAIR1 (Leukocyte associated immunoglobulin-like receptor 1)	−0.107	0.142	0.248	2.84 × 10^−7^	2206	90	2.91 × 10^−5^
CST3 (Cystatin C)	−0.086	0.071	0.160	3.04 × 10^−7^	2205	89	2.97 × 10^−5^
PRAP1 (Proline-rich acidic protein 1)	−0.132	0.097	0.238	3.34 × 10^−7^	2227	90	3.12 × 10^−5^
DNER (Delta and notch-like epidermal growth factor-related receptor)	0.086	−0.080	−0.164	3.82 × 10^−7^	2210	90	3.43 × 10^−5^
SORCS2 (Sortilin-related VPS10 domain containing receptor 2)	−0.098	0.104	0.202	7.13 × 10^−7^	2180	85	6.13 × 10^−5^
TNFRSF10A (Tumor necrosis factor receptor superfamily member 10A)	−0.091	0.093	0.180	7.54 × 10^−7^	2222	87	6.24 × 10^−5^
SCARB2 (Scavenger receptor class B member 2)	−0.087	0.071	0.154	7.87 × 10^−7^	2184	84	6.27 × 10^−5^
PGF (Placenta growth factor)	−0.069	0.066	0.135	1.08 × 10^−6^	2232	88	8.28 × 10^−5^
RNASE6 (Ribonuclease A family member K6)	−0.071	0.072	0.143	1.14 × 10^−6^	2216	89	8.48 × 10^−5^

NOR: normal, CP: chronic periodontitis, FDR: false discovery rate. Effect sizes were estimated from linear regression models adjusted for age, sex, and measurement date. The numbers of samples contributing to each protein (*n* NOR and *n* CP) may vary across proteins because samples with missing normalized protein expression (NPX) values or missing covariates were excluded on a per-protein basis after quality control. In addition, proteins and samples failing limit-of-detection and missingness filtering were removed prior to statistical analysis. False discovery rates (FDRs) were calculated using the Benjamini–Hochberg procedure.

## Data Availability

Availability of data and materials. The data generated in the present study may be requested from the corresponding author.
